# Maternal near miss and quality of maternal health care in Baghdad, Iraq

**DOI:** 10.1186/1471-2393-13-11

**Published:** 2013-01-16

**Authors:** Maysoon Jabir, Imad Abdul-Salam, Dhikra M Suheil, Wafa Al-Hilli, Sana Abul-Hassan, Amal Al-Zuheiri, Rasha Al-Ba'aj, Abeer Dekan, Özge Tunçalp, Joao Paulo Souza

**Affiliations:** 1 Baghdad Teaching Hospital, Baghdad, Iraq; 2 Department of Health and Biostatistics /Ministry of Health, Baghdad, Iraq; 3 Al-Yermouk Teaching Hospital, Baghdad, Iraq; 4 Al-Kadimeyah Teaching Hospital, Baghdad, Iraq; 5 Al-Elwiyah Maternity Teaching Hospital, Baghdad, Iraq; 6 Ibn Al-Bildi Hospital for Women and Children, Baghdad, Iraq; 7 Fatima Al-Zahraa' Maternity Hospital, Baghdad, Iraq; 8 Department of Population, Family and Reproductive Health, Johns Hopkins Bloomberg School of Public Health, 615 N Wolfe St, Baltimore, MD, 21205, USA; 9 Department of Reproductive Health and Research, World Health Organization, Geneva, Switzerland

**Keywords:** Maternal morbidity, Obstetric complications, WHO near-miss approach, Quality improvement, Developing countries, Baghdad

## Abstract

**Background:**

The maternal near-miss concept has been developed as an instrument for assisting health systems to evaluate and improve their quality of care. Our study aimed at studying the characteristics and quality of care provided to women with severe complications in Baghdad through the use of the World Health Organization (WHO) near-miss approach for maternal health.

**Methods:**

This is a facility-based, cross-sectional study conducted in 6 public hospitals in Baghdad between March 1, 2010 and the June 30, 2010. WHO near-miss approach was utilized to analyze the data in terms of indicators of maternal near miss and access to and quality of maternal care.

**Results:**

The maternal near-miss rate was low at 5.06 per 1,000 live births, while the overall maternal near miss: mortality ratio was 9:1. One third of the near-miss cases were referred from other facilities and the mortality index was the same for referred women and for in-hospital women (11%). The intensive care unit (ICU) admission rate was 37% for women with severe maternal outcomes (SMO), while the overall admission rate was 0.28%. Anemia (55%) and previous cesarean section (45%) were the most common associated conditions with severe maternal morbidity. The use of magnesium sulfate for treatment of eclampsia, oxytocin for prevention and treatment of postpartum hemorrhage, prophylactic antibiotics during caesarean section, and corticosteroids for inducing fetal lung maturation in preterm birth is suboptimum.

**Conclusions:**

The WHO near-miss approach allowed systematic identification of the roadblocks to improve quality of care and then monitoring the progress. Critical evidence-based practices, relevant to the management of women experiencing life-threatening conditions, are underused. In addition, possible limitations in the referral system result in a very high proportion of women presenting at the hospital already in a severe health condition (i.e. with organ dysfunction). A shortage of ICU beds leading to women taken care of without admission to ICU may also contribute to a high proportion of maternal deaths and organ dysfunction.

## Background

In 2000, the leaders of all United Nations Member States agreed that policies conducive to development and to the elimination of extreme poverty would be put in place in global scale. A set of goals has been established and many countries have done a substantial progress towards those goals, which became known as the Millennium Development Goals (MDGs) [1]. In Iraq, progress towards achievement of MDGs has been challenged by two major wars in the last two decades. On top of the unbearably high human cost, these wars have produced an important damage to the country’s infrastructure and affected various components of the health systems.

Social determinants and the health system performance play a major role in the occurrence of maternal deaths. One of the MDGs, reduction of maternal mortality is a robust indicator of development. In this context, even with the two wars, since 1990 there is a trend towards reduction of maternal mortality in Iraq. The World Health Organization estimates that in 1990, the maternal mortality in Iraq was around 93 deaths per 100,000 live births, while in 2008, it was estimated to be around 75 deaths per 100,000 live births [2]. Recent estimates are compatible with this trend [3], which is most likely due to the resilience of Iraqi people in keeping basic infrastructure and services functional under adverse conditions and, possibly, the substantial efforts for reconstruction have produced some visible effect.

Confidential enquiries and similar clinical audits of maternal deaths have been used to evaluate and improve the quality of maternal health care in many countries. However, as maternal deaths become less frequent or are found to be relatively rare in individual health facilities, the assessment of quality of care performed exclusively based on maternal deaths becomes more challenging. The concept of maternal near miss has been evolving during the past two decades as number of women dying from complications of pregnancy and childbirth is progressively decreasing in many countries, while the number of those with life-threatening complications who are treated and discharged home exceeds the number of those who die. Therefore, studying those women who nearly died but survived, identified as the near-miss cases, would give a better indication of care provided for those women who survived the near-miss event [4].

Overall, there had been three major approaches to the identification of near miss cases: 1) Clinical criteria related to a specific disease entity (i.e., pre-eclampsia, postpartum hemorrhage), 2) Management-based criteria (i.e., admission to ICU, need for a blood transfusion), or 3) Organ system dysfunction based criteria [5,6]. Depending on these different approaches, prevalence of near miss varies. According to a recent systematic review, prevalence rates of near miss varied between 0.6 and 14.98% for disease-specific criteria, between 0.04 and 4.54% for management-based criteria and between 0.14 and 0.92% for organ-based dysfunction based on Mantel criteria [7]. Women in resource-poor settings experience a higher prevalence in all these categories. However, due to wide variation in identification of cases as well as the variation within each category, it has not been possible to pool the data and make a summary estimate [4,7]. Currently, a maternal near-miss case is defined as *"a woman who nearly died but survived a complication that occurred during pregnancy, childbirth or within 42 days of termination of pregnancy”*[8]. In order to overcome these challenges, the maternal near-miss concept has been developed as an instrument for assisting health systems to evaluate and improve their quality of care. In recent years, WHO has developed a tool for evaluating the quality of maternal health care based on the near-miss concept [9].

In an attempt to standardize the identification of maternal near-miss events, the WHO working group for maternal mortality and morbidity classifications developed a consensus on maternal near-miss identification (Additional file 1: Table S1), which is based on two components [8]:

1. Identification of potentially life-threatening conditions, which may or may not be near-miss cases (i.e. specific complications such as severe preeclampsia and/or critical interventions such as blood transfusion).

2. Identification of near-miss cases based on organ system dysfunction and organ-dysfunction proxies including clinical, laboratory and management criteria.

Our study aims at assessing the prevalence and evaluating the management of severe maternal morbidity using the WHO near-miss approach in Baghdad, Iraq [9]. This is the first multi-center study conducted in the city of Baghdad to assess the near-miss cases in obstetric practice in Iraq.

## Methods

This is a facility-based, cross-sectional study conducted in six public hospitals in Baghdad between March 1, 2010 and the June 30, 2010. It consists of a near-miss criterion-based clinical audit implemented according the WHO near-miss approach for maternal health [6]. Selection criteria for the facilities included were 1) to be a public hospital and 2) to have more than one thousand deliveries per year according to the information provided by the health and biostatistics department in the Iraqi Ministry of Health. We included all of the hospitals fitting these criteria in Baghdad: a total of six. These public hospitals were distributed all over Baghdad city to serve a population of approximately seven millions. Three of these hospitals were general hospitals with obstetric units and the rest were major maternity hospitals. Four out of six hospitals had intensive care units (ICUs), although the remaining two hospitals had a "close observation unit" to monitor and treat women with post-operative and post-delivery complications, run by specialized obstetricians. These hospitals receive referrals from midwives, health centers, private hospitals as well as unbooked patients (patients self-referring themselves to the hospitals). No fees are paid by patients for the services provided.

A study coordinator among the hospital coordinators was designated in each of these six facilities and the overall study coordination was performed by the Centre of Training and Human Development in the Iraqi Ministry of Health. These six selected study coordinators were trained on a two-day course in January 2010 on identifying severe life-threatening conditions, maternal near-miss events and deaths, and how to implement data collection.

The WHO consensus on maternal near-miss definition was used to define the cases [8]. Definitions and abbreviations used in this paper are found in Table 1. Data were collected on a daily basis by the coordinators using hospital records or staff interviews and the forms were filled while the women were still in the hospital. Cases were defined according to potentially life-threatening conditions including severe postpartum hemorrhage, severe preeclampsia, eclampsia, sepsis and ruptured uterus, whereas organ or system failure depending on certain clinical criteria, laboratory markers and management proxies were used to identify the near-miss cases among potentially life-threatening conditions (Additional file 1: Table S1). The maternal outcome, gestational age and neonatal outcome were also collected during the hospital stay or by the 7th day postpartum, whichever came first.

**Table 1:** **Maternal near-miss terminology and indicators**[8,9]

	
**Maternal Near Miss (MNM)**	A woman who nearly died but survived a complication that occurred during pregnancy, childbirth or within 42 days of termination of pregnancy.
**Maternal Death (MD)**	Death of a woman while pregnant or within 42 days of termination of pregnancy or its management, but not from accidental or incidental causes.
**Live Birth (LB)**	The birth of an offspring, which breathes or shows evidence of life.
**Severe maternal outcome (SMO)**	A life-threatening condition (i.e. organ dysfunction), including all maternal deaths and maternal near-miss cases.
**Women with life-threatening conditions (WLTC)**	All women who either qualified as having maternal near miss or who died. It is the sum of maternal near miss and maternal deaths.
**Maternal Near Miss Ratio (MNMR)**	The number of maternal near miss cases per 1,000 live births.
**Severe Maternal Outcome Ratio (SMOR)**	The number of women with life threatening conditions per 1,000 live births. This indication gives an estimation of the amount of care and resources that would be needed in an area or facility.
**Maternal Near Miss Mortality Ratio:**	The ratio between maternal near-miss cases and maternal deaths. Higher ratios indicate better care.
**Mortality Index**	The number of maternal deaths divided by the number of women with life threatening conditions, expressed as a percentage. The higher the index the more women with life-threatening conditions die (low quality of care), whereas the lower the index the fewer women with life-threatening conditions die (better quality of care).
**Perinatal outcome indicators**	(e.g. perinatal mortality, neonatal mortality or stillbirth rates) in the context of maternal miss could be useful to complement the quality of care evaluation.
**Hospital Access Indicators:**	
The following indicators are used to explore the access to the facility in terms of functioning referral systems.	· *SMO12:* Cases presenting the organ dysfunction or maternal death within 12 hours of hospital stay
	· *Proportion of SMO12 cases among all SMO cases*
	· Proportion of SMO12 cases coming from other facilities
	· *SMO12 mortality index:* The number of SMO12 cases divided by the number of all SMO cases expressed as a percentage.
**Intra-hospital Care:**	
The following indicators are used to explore access to quality care in the facility:	· *Intra-hospital SMO:* Cases presenting the organ dysfunction or maternal death after 12 hours of hospital stay.
	· *Intra-hospital SMO rate (per 1000 live births):* The number of intra-hospital SMO cases per 1000 live births.
	· *Intra-hospital mortality index:* The number of intra-hospital SMO cases divided by the number of all SMO cases expressed as a percentage.
	· *ICU admission rate:* The number of women admitted to ICU among total number of womengiving birth.
	· *ICU admission rate among women with SMO:* The number of women with SMO divided by the ICU admissions among total number of women giving birth.
**Process Indicators:**	
The following indicators are used to assess the coverage of selected evidence-based interventions used for prevention and treatment of the main causes of maternal deaths.	· *Prevention of postpartum hemorrhage:* The number of women who received a single dose of oxytocin divided by the number of all women giving birth (vaginal delivery + cesarean section)
	· *Treatment of severe postpartum hemorrhage:* The number of women with severe PPH who received therapeutic oxytocin divided by the number of all women with postpartum hemorrhage.
	· *Eclampsia:* The number of women with eclampsia who received magnesium sulfate divided by the number of all women with eclampsia.
	· *Prevention of severe systemic infections/sepsis*: The number of women having a cesarean section and receiving prophylactic antibiotics divided by the number of all women having cesarean sections.
	· *Treatment of severe infections and sepsis:* The number of women with severe systemic infections or sepsis who received IV antibiotics divided by the number of all women with severe systemic infections or sepsis.
	· *Fetal lung maturation*: The number of women having a live birth after 3 hours of hospital stay and receiving corticosteroids for fetal lung maturation divided by all women having a live birth after 3 hours of hospital stay.

Using the data collected, various indicators have been calculated. In line with maternal mortality ratio, maternal near-miss ratio was the number of near-miss cases per 1000 live births. Moreover, maternal near-miss mortality ratio, which is the ratio between maternal near-miss cases and maternal deaths, was calculated. For this indicator, higher ratios indicate better care, meaning more women survived as a near miss rather than becoming maternal deaths. Also, mortality index was calculated, where the number of maternal deaths was divided by the number of women with life-threatening conditions (maternal near miss and maternal deaths) and was expressed as a percentage. Higher indices indicate that more women with life-threatening conditions die (low quality of care), whereas lower indices signify better quality of care.

Access to hospital and intra-hospital care were assessed by the proportion of near-miss cases and maternal deaths presenting within 12 hours of hospital stay versus after 12 hours, the latter indicating the quality of care provided within the hospital. Intensive care unit (ICU) use among our study population was collected as well.

We also collected data on the coverage of selected evidence-based interventions used for prevention and treatment of the main causes of maternal deaths. This was part of a criterion-based clinical audit approach used to assess the quality of care and included interventions related to the prevention and treatment of postpartum hemorrhage, severe preeclampsia and eclampsia, use of antibiotics for infection prophylaxis during caesarean section (C-section) and treatment of sepsis. In addition, we measured the use of corticosteroids for fetal lung maturity.

Data were sent monthly to the country coordinator and subsequently entered into an online data entry system. This online data management system was based on the Google platform and data entered in the online form were stored in an online spreadsheet, which incorporated a comprehensive set of consistency rules to provide concurrent data quality check. The inconsistencies that were identified generated queries to the study coordinators. The standard data tables based on the WHO near-miss approach was automatically generated using Microsoft Excel as the data were entered. The online data entry system was password protected.

The study was approved by the ethics committee of the local supervising committee of the Arab Board for Health Specializations (ABHS). All data were obtained from medical records and did not identify participants, therefore each site was granted a waiver of individual informed consent.

## Results

During the four-month data collection period in the six study facilities, overall there were 25,472 live births, 212 women with potentially life threatening conditions and 145 severe maternal outcomes (129 near-miss cases and 16 maternal deaths). Our results will be presented as suggested in the WHO handbook on the near-miss approach for maternal health [9].

Cases were identified according to underlying causes of morbidity and organ system dysfunction in Tables 2 and 3, respectively. Most common organ dysfunctions reported among near-miss cases were cardiovascular dysfunction followed by uterine dysfunction leading to hysterectomy, 55.8 and 53.5, respectively (Table 2). It should be noted that almost 50% of the women with maternal near-miss morbidity had multiple organ dysfunction. Mortality index was highest for renal dysfunction, 40%. Maternal near-miss mortality ratio was highest for uterine dysfunction (11:1), followed by cardiovascular and coagulation/ hematologic disorders (10:1). Of all the women with potentially life-threatening conditions, 181 women (85.4%) underwent the following critical interventions: 118 (55.7%) women used blood products, 78 (36.8%) women had laparotomy and 75 (34.4%) women were admitted to ICU.

**Table 2:** Morbidity conditions leading to inclusion in a sample of women with potentially life-threatening conditions and severe maternal outcomes

	**n**	**%**
**Women with potentially life-threatening conditions**	212	100.00%
Women with severe complications	174	82.08%
Severe postpartum hemorrhage	84	39.62%
Severe Preeclampsia	4	1.89%
Eclampsia	43	20.28%
Sepsis or severe systemic infection	10	4.72%
Ruptured uterus	29	13.68%
Other complications associated with severe maternal outcome	22	10.38%
Women undergoing critical Interventions	181	85.38%
Use of blood products	118	55.66%
Interventional radiology (uterine artery embolization)	2	0.94%
Laparotomy	85	40.09%
Admission to Intensive Care Unit	73	34.43%
**Organ dysfunction in maternal near-miss cases**	129	100.00%
Cardiovascular dysfunction	72	55.81%
Respiratory dysfunction	30	23.26%
Renal dysfunction	6	4.65%
Coagulation/hematologic dysfunction	30	23.26%
Hepatic dysfunction	5	3.88%
Neurologic dysfunction	23	17.83%
Uterine dysfunction/hysterectomy	69	53.49%
Multiple organ dysfunction	64	49.61%
**Organ dysfunction in maternal deaths**	16	100.00%
Cardiovascular dysfunction	7	43.75%
Respiratory dysfunction	8	50.00%
Renal dysfunction	4	25.00%
Coagulation/hematologic dysfunction	3	18.75%
Hepatic dysfunction	0	0.00%
Neurologic dysfunction	4	25.00%
Uterine dysfunction/hysterectomy	6	37.50%
Unspecified organ dysfunction	1	6.25%
Multiple organ dysfunction	9	56.25%

**Table 3:** Underlying causes of potentially life-threatening conditions and severe maternal outcomes

	**Women with potentially life-threatening conditions**		**Maternal near-miss cases**		**Maternal deaths**	
	N=	212	N=	129	N=	16
	n	%	n	%	n	%
**Underlying causes**						
Pregnancy with abortive outcome	44	20.75%	6	4.65%	1	6.25%
Obstetric Hemorrhage	99	46.70%	85	65.89%	7	43.75%
Hypertensive disorders	49	23.11%	27	20.93%	4	25.00%
Pregnancy-related infection	9	4.25%	4	3.10%	1	6.25%
Other obstetric disease or complication	28	13.21%	22	17.05%	1	6.25%
Medical/Surgical/Mental disease or complication	38	17.92%	26	20.16%	5	31.25%
Unanticipated complications of management	14	6.60%	11	8.53%	1	6.25%
Coincidental conditions	10	4.72%	8	6.20%	2	12.50%
Unknown	4	1.89%	3	2.33%	1	6.25%
**Contributory causes / associated conditions**						
Anemia	96	45.28%	71	55.04%	8	50.00%
HIV infection	0	0.00%	0	0.00%	0	0.00%
Previous caesarean section	74	34.91%	58	44.96%	7	43.75%
Prolonged / obstructed labor	28	13.21%	21	16.28%	3	18.75%

Underlying causes were similar among potentially life-threatening conditions, near-miss cases and maternal deaths, most common being severe postpartum hemorrhage followed by hypertensive disorders (Table 3). Associated conditions have also been assessed and the most common reported conditions were anemia and previous C-section, 55% and 44.5% among near-miss women, respectively. Despite the small numbers, the highest maternal near-miss mortality ratio among these underlying causes was observed among women with pregnancy-related infections (25%).

Majority of women with potentially life-threatening conditions, near-miss cases and maternal deaths had C-sections, 51%, 61% and 69%, respectively. Neonatal outcomes (preterm births, stillbirths and perinatal deaths) were worst among maternal deaths and comparable between women with potentially life-threatening conditions and maternal near misses (Table 4). Overall, there were no women with complete abortion reported and ectopic pregnancy was highest among women with potentially life-threatening conditions (18.4%) followed by maternal deaths (6.3%).

**Table 4:** End of pregnancy and pregnancy outcomes in our study population

	**Potentially life-threatening conditions**		**Maternal near-miss cases**		**Maternal deaths**	
	N=	212	N=	129.	N=	16
	n	%	n	%	n	%
**End of pregnancy**						
Vaginal delivery	46	21.70%	37	28.68%	3	18.75%
Caesarean Section	107	50.47%	78	60.47%	11	68.75%
Complete abortion	0	0.00%	0	0.00%	0	0.00%
Curettage / vacuum aspiration	7	3.30%	5	3.88%	0	0.00%
Medical methods for uterine evacuation	0	0.00%	0	0.00%	0	0.00%
Laparotomy for ectopic pregnancy	39	18.40%	1	0.78%	1	6.25%
Other	4	1.89%	2	1.55%	0	0.00%
Unknown	2	0.94%	1	0.78%	0	0.00%
Women still pregnant at hospital discharge or death	7	3.30%	5	3.88%	1	6.25%
Caesarean section rate*	n/a	69.93%	n/a	67.83%	n/a	78.57%
**Preterm births**	57	37.25%	42	36.52%	8	57.14%
**Stillbirths**	32	20.92%	23	20.00%	6	42.86%
Perinatal deaths**	65	42.48%	50	43.48%	7	50.00%

*Caesarean deliveries divided by all deliveries / **fetal deaths + intra-hospital early neonatal mortality.

The total maternal mortality ratio for the hospitals was 62.8 per 100,000 live births. Severe maternal outcomes and near-miss indicators can be found in Table 5. The maternal near-miss ratio (MNMR) and severe maternal outcome ratio (SMOR) were 5.06 and 5.69 per 1,000 live births, respectively (Table 5).

**Table 5:** Severe maternal outcomes and near miss indicators

**All live births in the population under surveillance**	25,472
**Severe maternal outcomes (SMO) cases (n)**	145
Maternal Deaths (n)	16
Maternal near miss cases (n)	129
**Overall near-miss indicators**	
Severe Maternal Outcome ratio (per 1000 live births)	5.69
Maternal near miss incidence ratio (per 1000 live births)	5.06
Maternal near miss mortality ratio	9:1
Mortality index	11.03%
**Hospital access indicators**	
SMO cases presenting the organ dysfunction or maternal death within 12 hours of hospital stay (SMO12) (n)	127
Proportion of SMO12 cases among all SMO cases	87.59%
Proportion of SMO12 cases coming from other health facilities	34.65%
SMO12 mortality index	11.02%
**Intra-hospital care**	
Intra-hospital SMO cases (n)	18
Intra-hospital SMO rate (per 1000 live births)	0.71
Intra-Hospital mortality index	11.11%

### Access to Hospital and Care Indicators

Of the 145 cases with severe maternal outcomes (near miss and maternal deaths), 127 cases (87.6%) presented with the organ dysfunction or maternal death within the first 12 hours of hospital admission and 34% of these cases were referred from other facilities (Table 5). The mortality indices for the first 12 hours of hospital admission and after 12 hours (intra-hospital) are 11.02% and 11.11%, respectively.

Among all the women giving birth at our study facilities, the overall ICU admission rate was 0.28%, whereas ICU admission rate among women with severe maternal outcomes was 37.2% and proportion of maternal deaths occurred with ICU admission was 50% (Table 6).

**Table 6:** Intensive care use in our study population

**All women giving birth**	**25,841**
ICU admission rate	0.28%
ICU admission rate among women with SMO	37.24%
SMO rate among women admitted to ICU	73.97%
Proportion of maternal deaths occurred without ICU admission	50.00%

### Process Indicators

Among women with severe maternal outcomes, oxytocin was used in 86% of the women for the prevention of postpartum hemorrhage, whereas only 68% of the women with severe PPH received oxytocin as a treatment agent among other treatment regimens. In 93% of the eclampsia cases an anticonvulsant was used and MgSO4 was used in 67% of these cases. Antibiotics were used for all women with an established infection. In case of caesarean section, 61% of women received prophylactic antibiotics intra-operatively and the rest 39% were given antibiotics post-operatively. Corticosteroids for fetal lung maturity were used in only 55% of pregnant women eligible for this type of treatment (Table 7).

**Table 7:** Process outcome indicators related with specific conditions among women with severe maternal outcomes (maternal near miss and maternal deaths)

	**n**	**%**
**Prevention of postpartum hemorrhage**		
Target population: women giving birth in health facilities	132	100.00%
Oxytocin	114	86.36%
Any uterotonic (including oxytocin)	115	87.12%
**Treatment of severe postpartum hemorrhage**		
Target population: women with severe PPH	84	100.00%
Oxytocin	57	67.86%
Ergometrine	52	61.90%
Misoprostol	35	41.67%
Other uterotonics	3.	3.57%
Any of the above uterotonics	57	67.86%
Tranexamic acid	6	7.14%
Removal of retained products	16	19.05%
Balloon or condom tamponade	1.	1.19%
Artery ligation	4	4.76%
Hysterectomy	50	59.52%
Abdominal packing	4	4.76%
Proportion of cases with SMO	80	95.24%
Mortality	6.	7.14%
**Anticonvulsants for Eclampsia**		
Target population: women with eclampsia	43	100.00%
Magnesium sulfate	29	67.44%
Other anticonvulsant	22	51.16%
Any anticonvulsant	40	93.02%
Proportion of cases with SMO	26	60.47%
Mortality	4	9.30%
**Prevention of caesarean section related infection**		
Target population: women undergoing caesarean section	107	100.00%
Prophylactic antibiotic during caesarean section	65	60.75%
**Treatment for sepsis**		
Target population: women with sepsis	10	100.00%
Parenteral therapeutic antibiotics	10	100.00%
Proportion of cases with SMO	6	60.00%
Mortality	2	20.00%
**Ruptured uterus**		
Target population: women with ruptured uterus	29	100.00%
Laparotomy	28	96.55%
Laparotomy after 3 hours of hospital stay	5	17.24%
Proportion of cases with SMO	24	82.76%
Mortality	3	10.34%
**Preterm birth**		
Target population: women having a preterm delivery after 3 hours of hospital stay	18	100.00%
Corticosteroids for fetal lung maturation	10	55.56%

## Discussion

This study shows that in urban Iraq, the prevalence of maternal deaths and near-miss cases is relatively low. Despite that, this study also highlights some opportunities to improve care, both at the facility level and at the organization of care / health system level. Some evidence-based practices, relevant to the management of women experiencing life-threatening conditions, are underused. In addition, possible limitations in the referral system result in a very high proportion of women presenting at the hospital already in a severe health condition (i.e. with organ dysfunction). A shortage of ICU beds may also contribute to a high proportion of maternal deaths and organ dysfunction taking care without admission to ICU.

Comparing the major causes of near-miss cases and maternal deaths, obstetric hemorrhage and hypertension were the most common underlying causes of severe maternal outcomes, which is comparable to other studies in developing countries [10-12]. In our study, anemia is found in more than 50% of women with severe maternal outcomes, and when compared with population level data among Iraqi pregnant women (37.9%), this is a statistically significant difference, underlining the vulnerable status of this sub-population [13].

In under-resourced settings there is a need to separate the near-miss cases on arrival to hospital from those that develop in the hospital setting as the former indicates a failure in access to the facilities and/or to the referral chain where such hospitals would need adequate resources and organization to deal with such emergencies [14]. In our study two thirds of women with SMO developing within the first twelve hours of admission were admitted without referrals, which may indicate an issue in the referral system and/or the detection of pregnant women with life-threatening complications outside these hospitals due to delay in seeking care or reaching care. However it should be noted that in our study, the overall mortality index is very similar between women with severe maternal outcomes who were referred versus in-hospital patients. This is in contrast to a similar study where SMO was relatively higher among referred women [12].

One of the key principles of effective management of complications related to pregnancy and childbirth is matching the level of care to the severity of the clinical conditions. Women presenting complications may require different levels of care, from basic obstetric care to intensive care, including in this continuum comprehensive obstetric care and surgery. Most of the women presenting organ dysfunction would be more appropriately managed at the ICU level [15]. The low ICU admission rates observed in this study suggests an important shortage of ICU beds, which is corroborated by the substantial proportion of women experiencing organ dysfunction or dying without access to ICU bed. In addition, it should be noted that two of the six hospitals had “close observation units” instead of a proper ICU. Overall, the provision of adequate critical care, with appropriate staffing, equipment and management strategies can contribute to a better outcome among women with life-threatening conditions [15].

The use of a criterion-based clinical audit methodology within WHO near-miss approach revealed opportunities to improve care, where a target population with a clear indication of an effective intervention is identified and then the use of this specific intervention is assessed. Among the hospitals in our study, magnesium sulfate for treatment of eclampsia, oxytocin for prevention and treatment of postpartum hemorrhage, prophylactic antibiotics during cesarean section, and corticosteroids for inducing fetal lung maturation in preterm birth were all underused. Of note, there were 5 cases of ruptured uterus that had the laparotomy being performed after three hours of hospital stay, suggesting an intra-hospital delay in the management of obstructed labor.

This study has several strengths that deserve noting. This is the first study assessing the quality of care in the Iraqi facilities using the recent WHO maternal near-miss definition and criteria. We collaborated with the Iraqi Ministry of Health and by conducting the study with the participation of the hospital staff and local capacity strengthening, we aimed to create a long lasting surveillance and quality improvement mechanism in those hospitals, which can be replicated in other resource-poor settings. There are some limitations as well. Our study was conducted in only six hospitals in Baghdad; therefore our results cannot be generalized to the overall country. Also, we presented our data in aggregate, however an in-depth analysis would be more beneficial to assess the causes and contributory factors in individual hospitals to improve quality of care. More importantly this manuscript only describes our work to evaluate the quality of care and our subsequent findings, but does not cover the ongoing efforts to improve care based on these results. Also, it should be noted that we only collected data up to 7th day postpartum, whereas the definition of near miss includes cases up to 42 days.

## Conclusions

The use of the WHO maternal near-miss approach enabled the identification of important roadblocks to improve quality of maternal care in Baghdad, Iraq. Our results suggest that severe maternal outcomes can be potentially reduced by fostering the use of evidence-based interventions for life-threatening complications, improving referral systems, and optimizing the use of critical care.

## Competing interests

The authors declare that they have no competing interests.

## Authors’ contributions

MJ had the idea of testing the WHO Maternal near-miss approach in Iraq. MJ and JPS developed the study protocol and data collection processes. MJ, IA, DMS, WA, SA, AA, RA, AD implementation the Study in Iraq and provided critical input during the study development, interpretation of findings and reporting of the study results. MJ, OT and JPS drafted the manuscript and lead the interpretation and presentation of the results. All authors contributed to drafting and revising the manuscript and have read and approved the final version.

## Pre-publication history

The pre-publication history for this paper can be accessed here:

http://www.biomedcentral.com/1471-2393/13/11/prepub

## Supplementary Material

Additional file 1 **Table S1.** Criteria to identify potentially life-threatening conditions and near miss [9]. Click here for file
