# Supplementary material for: Maternal near miss and quality of maternal health care in Baghdad, Iraq
Source: BMC Pregnancy Childbirth. 2013 Jan 16;13:11. doi: 10.1186/1471-2393-13-11 (PMC3558361; doi:10.1186/1471-2393-13-11)
Supplement: Additional file 1 — Table S1. Criteria to identify potentially life-threatening conditions and near miss [9]. [file 1471-2393-13-11-S1.doc]

**Supplemental Table 1: Criteria to identify potentially life-threatening conditions and near miss [**[**9**](#_ENREF_9)**]**

| POTENTIALLY LIFE-THREATENING CONDITIONS |
| --- |
| Severe complications  1. Severe postpartum hemorrhage: genital bleeding after delivery, with at least one of the following perceived abnormal bleeding (1000 mL or more) or any bleeding with hypotension or blood transfusion.  2. Severe preeclampsia: Persistent systolic blood pressure of 160 mmHg or more or a diastolic blood pressure of 110 mmHg; proteinuria of 5 g or more in 24 hours; oliguria of <400 ml in 24 hours; and HELLP syndrome or pulmonary edema. Excludes eclampsia.  3. Eclampsia: generalized fits in a patient without previous history of epilepsy. Includes coma in preeclampsia.  4. Sepsis or severe systemic infection: presence of fever (body temperature > 38°C), a confirmed or suspected infection (e.g. chorioamnionitis, septic abortion, endometritis, pneumonia), and at least one of the following- heart rate>100, respiratory rate>20, leukopenia (white blood cells <4000), leukocytosis (white blood cells >12 000)  5. Ruptured uterus: ruptured uterus during labour |
| Critical Interventions  1. Use of blood products  2. Laparotomy (including hysterectomy, excluding C-section)  3. Admission to Intensive Care Unit/recovery room >=6 hours |
| NEAR-MISS CRITERIA |
| Clinical Organ Dysfunction  1. Acute cyanosis  2. Gasping  3. Respiratory rate >40 or <6 bpm  4. Shock  5. Cardiac Arrest  6. Oliguria non-responsive to fluids or diuretics  7. Any loss of consciousness lasting >12 hours  8. Stroke  9. Uncontrollable fit/status epilepticus  10. Global paralysis  11. Jaundice in the presence of pre-eclampsia |
| Laboratory markers of organ dysfunction  12. O2 saturation <90% for more than 60 min  13. PaO2/FiO2<200 mmHg  14. Creatinine>300umol/ml or >3.5 mg/dL  15. Bilirubin>100umol/L or >6.0 mg/dL  16. pH<7.1  17. Lactate > 5mEq/L  18. Acute thrombocytopenia (<50,000 platelets) |
| Management-based proxies  19. Hysterectomy following infection or hemorrhage  20. Use of continuous vasoactive drugs  21. Cardio-pulmonary resuscitation  22. Dialysis for acute renal failure  23. Any non-anesthetic intubation or ventilation  24. Transfusion of >5 units of blood or red cells |
